# Therapeutic Liquid Eutectic Solvents in Lipid Nanoparticles as Drug Vehicles—A Proof of Concept

**DOI:** 10.3390/ijms242115648

**Published:** 2023-10-27

**Authors:** Joana Gonçalves, Cláudia Marques, Cláudia Nunes, Mafalda Sarraguça

**Affiliations:** LAQV/REQUIMTE, Faculdade de Farmácia, Universidade do Porto, Rua de Jorge Viterbo Ferreira, 228, 4050-313 Porto, Portugal; joana.goncalves@umu.se (J.G.); up201905182@edu.fc.up.pt (C.M.)

**Keywords:** tuberculosis, ethambutol, therapeutic deep eutectic solvents, nanostructured lipid carriers, stability

## Abstract

Tuberculosis is an infectious disease caused by the bacterial complex *Mycobacterium tuberculosis*. Despite the decline in the incidence and mortality of this disease over the years, the emergence of new strains of tuberculosis resistant to existing tuberculostatic drugs is currently one of the largest public health problems. The engineering and development of new drugs is a complex process; therefore, the modification and enhancement of the drugs already marked is a better and faster solution. Ethambutol (ETB) is an antimycobacterial drug used to treat tuberculosis; however, it is highly hygroscopic and is sparingly soluble in water. Therapeutic Deep Eutectic Solvents (THEDESs) are known to improve drug solubility, permeability, and hygroscopicity, among others. In this study, three THEDESs of ETB were synthesized with sucralose, glucose and glycerol and then encapsulated in nanostructured lipid carriers to improve their stability. This work is a proof of concept on the possibility of encapsulating the THEDESs, and results show that the encapsulation of ETB is possible, yielding formulations with a loading capacity superior to 8.5% and able to incorporate THEDESs and not just ETB.

## 1. Introduction

With the increase in new reported cases of drug resistance to first-line drugs to treat tuberculosis (TB) like rifampicin, ethambutol, pyrazinamide, isoniazid, or rifabutin, and the surge of new resistant strains of TB, the development of new drugs or the enhancement of the existing ones is necessary [1].

Instead of engineering and developing new drugs that require new clinical trials, a longer process and increased costs, the improvement of therapies already used and tested can be more beneficial. Modification of the drug’s form or formulation, the use of different dosages or modifying the administration, assessment of new combinations, improvement of the hydrophilicity, and many more aspects can be a way of updating a drug and making it more effective [2].

TB is caused by Koch’s bacillus, also known as *Mycobacterium tuberculosis*. The TB epidemic reached its peak during the 18th and 19th centuries, and in 1993, the World Health Organization (WHO) declared TB a global crisis. In 2021, the WHO estimated 10.6 million new cases, of which 1.6 million resulted in death. TB is the 13th leading cause of death and the 2nd leading infectious killer after COVID-19. TB is present in all countries and age groups; however, it is a preventable and curable disease [3]. Rifampicin was the first drug used to treat TB, and in the 1970s, the standard quadruple anti-tuberculosis regimen, still used today, was implemented [4].

Ethambutol (ETB) is one of the first-line drugs for the treatment of TB; (S,S)-ethambutol is the therapeutically active stereoisomer and it is formulated in the form of dihydrochloride salt (ETH), (S,S)-ETH [5]. ETB action mechanism interferes with the mycobacterial wall synthesis inhibiting the arabinosyl transferase, which affects the synthesis of arabinogalactan and lipoarabinomannan [5,6]. Its ability to alter the permeability of the cell wall allows the passage of other antimicrobial drugs [6]. When the resistance to isoniazid is present, ETB also helps to prevent the emergence of rifampicin resistance [7].

One of the main concerns with the use of (S,S)-ETH lies with its high hygroscopicity, leading to the increased degradation of isoniazid and rifampicin by providing an acidic environment, resulting in the loss of drug efficiency upon storage [5]. In order to avoid drug–drug interactions and water uptake, (S,S)-ETH, isoniazid and pyrazinamide are coated separately with polymers and then are blended all together with rifampicin, formulating the final product. To deal with and overcome the mentioned disadvantageous effects of (S,S)-ETH, many studies have surfaced dealing with the development of multicomponent crystal forms for this drug [8,9]. In this work, a different approach is used with the development of liquid multicomponent forms in the form of Deep Eutectic Solvents (DESs).

DESs are a new generation of solvents currently gaining widespread scientific and technological attention as low-cost and greener alternatives to organic solvents and ionic liquids [10]. Eutectic mixture occurs when two or more components react in a defined stoichiometric proportion forming hydrogen interactions with each other and producing a mixture with a melting temperature below the melting temperature of the initial components [11]. DESs are eutectic mixtures that deviate from the ideal theoretic behavior and have a melting point depression to such an extent that they can be liquid at room or body temperature [12]. DESs can be formed with non-toxic and biodegradable materials and have physical–chemical properties like ionic liquids, such as low flammability, low vapor pressure, thermal stability, high viscosity due to strong hydrogen bonds, among others. However, DESs have some differences from ionic liquids such as the preparation methods, low or no toxicity, and biodegradability [10,13,14,15]. In addition, DESs are achieved with 100% atomic savings and without the formation of by-products [16,17].

Therapeutic Deep Eutectic Solvents (THEDESs) are DESs with an active pharmaceutical ingredient (API) as one of the constituents. The exploitation of THEDESs by integrating an API in the system can be used to modulate solubility, permeability, and stability, among other properties, and therefore improve the bioavailability of drugs [11,12,18]. A THEDES of ethambutol, citric acid and water was synthesized by Santos et al. [18]. The authors observed an increase in the solubility and permeability when compared with the crystalline ethambutol.

The use of nanocarriers to encapsulate drugs offers advantages such as protection of the drug from gastrointestinal degradation, improved circulation half-life, active and passive targeting of specific intestinal cells, reduced systemic side effects, codelivery of multiple drug combinations using a single nanocarrier, and the possibility to modulate drug release [19].

Lipid-based nanocarriers seem to have the most suitable features to load poorly soluble drugs since they may offer high drug loading, present suitable stability and are feasible for scale-up compared with other colloidal carriers [19]. Nanostructured Lipid Carriers (NLCs) are drug delivery systems composed of a solid lipid and a liquid lipid as a core matrix for the apprehension of the drug, in which the final product is still solid. A surfactant is also used to prevent aggregation phenomena and stabilize the lipid in aqueous phases [20,21]. NLCs have a larger loading capacity and prevent the premature expulsion of the drug, providing long-term stability when compared with other lipidic nanocarriers. This happens due to the chemical differences between the two lipids, which leads to imperfections inside the matrix improving the entrapment capacity [19]. The components of these nanoparticles are Generally Recognized as Safe (GRAS) and therefore can be used for oral administration [22].

In this study, THEDESs of ethambutol with sucralose, glucose and glycerol were encapsulated in NLCs to improve the stability of the THEDESs. To the best of our knowledge, this is the first study in which the encapsulation of THEDESs in lipidic nanoparticles is performed. This work can be considered as a proof of concept on the capability of NLCs to encapsulate the multicomponent THEDESs instead of the API alone.

Results showed that this approach is valid for further developments with encapsulation percentages around 45% and a loading capacity superior to 8.5%, which is a high value for NLC formulations.

## 2. Results and Discussion

**Figure 1:**
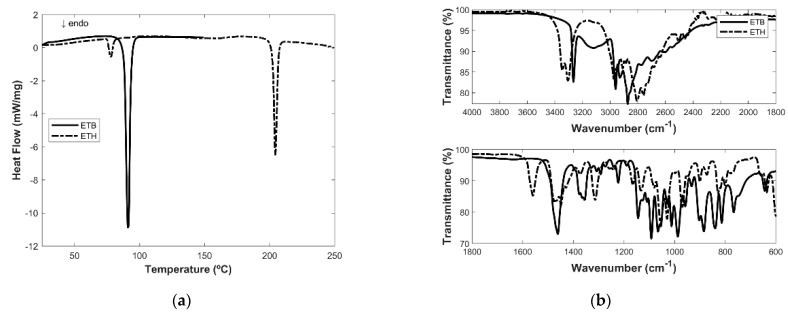
(**a**) Thermogram of ETB and ETH, (**b**) MIR spectra of ETB and ETH.

MIR spectroscopy and DSC analysis are used as characterization techniques to confirm that all the ETH is converted to ETB in the extraction procedure that is performed (Figure 1).

The thermogram of ETH (Figure 1a) shows a first endothermic peak with an onset of 75.2 °C and enthalpy change of −22.93 J/g correspondent to a solid–solid transition and a second endothermic event at 202.2 °C with an enthalpy change of −133.0 J/g correspondent to the melting point. The thermogram of ETB (Figure 1a) is clearly different with a single sharp endothermic peak at 88.3 °C corresponding to the melting with an enthalpy change of −262.7 J/g.

The MIR spectra for both components can be seen in Figure 1b. The spectrum of ETB presents a strong signal at 3264 cm^−1^ corresponding to the signal of the secondary amine. On the other hand, the spectrum of ETH shows a signal at 2972 cm^−1^ corresponding to the protonated amine and does not show the signal of the secondary amine.

Both techniques confirm that the extraction procedure is successful, and all hydrochloride salt of ethambutol reduces to its base form.

**Figure 2:**
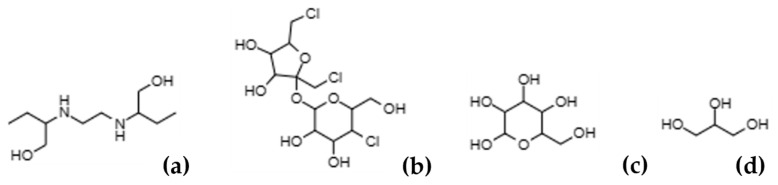
Structures of (**a**) ethambutol, (**b**) sucralose, (**c**) glucose and (**d**) glycerol.

The extracted ETB is used to synthesize the THEDESs with sucralose (SCE), glucose (GLU) and glycerol (GLY) (Figure 2). In these THEDESs, ETB is the hydrogen bond acceptor (HBA), and the sugars and alcohol are the hydrogen bond donor (HBD). Additionally, water is added to all THEDESs. The final molar ratios for each THEDES are ETB:SCE:H_2_O (2:1:8), ETB:GLU:H_2_O (2:1:8) and ETB:GLY:H_2_O (2:1:10).

All three THEDESs form a clear liquid; however, the THEDES with GLY is the less stable, starting to crystalize sooner after it reaches room temperature. This is not considered a problem since the encapsulation of the THEDESs in the nanostructured lipid carriers (NLCs) is performed at 70 °C, a temperature in which all THEDESs are liquid.

NLC of all three THEDESs are prepared and physiochemically characterized. Their main features are presented in Table 1.

The hydrodynamic radius of NLC encapsulated with THEDESs increases significantly compared with that of unloaded NLC. The polydispersity index (PDI) of the formulations is above 0.2, meaning that the formulations are more heterogeneous than the unloaded ones, but still have low variability considering the high shear homogenization and the ultrasound synthesis method. Furthermore, the zeta potential modulus also increased significantly. Taken together, these analyses point to a differential structure of NLC when loaded with THEDESs. Particularly, the differences in zeta potential can be correlated to the location of THEDESs at the surface of NLC, either adsorbed or linked. Zeta potential values higher than |45| mV ensure the stability of the formulations and reveal that they do not tend to form aggregates, since they are superior to the reference value for stability of the formulations of |30| mV [23]. Although the encapsulation efficiency (EE) is not so high, about 40%, the loading capacity is quite high for lipid-based delivery systems with values near 9% [24].

The THEDESs and lyophilized NLCs encapsulated with the THEDESs are characterized by DSC analysis and MIR spectroscopy.

**Figure 3:**
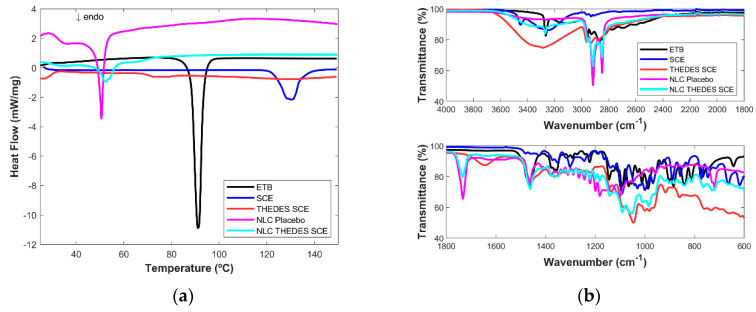
(**a**) DSC thermograms and (**b**) MIR spectra of ethambutol (ETB), sucralose (SCE), ethambutol THEDES with sucralose (THEDES SCE), NLCs placebo and the NLCs encapsulated with THEDES (NLC THEDES SCE).

The DSC thermograms for the THEDESs have very little information since no major transitions occur. For the THEDES SCE (Figure 3a), the only visible peak is an endothermic transition, which is very small, between 60 °C and 80 °C, maybe due to the fusion of some ETB that is not in THEDES form. However, this is not very clear.

**Figure 4:**
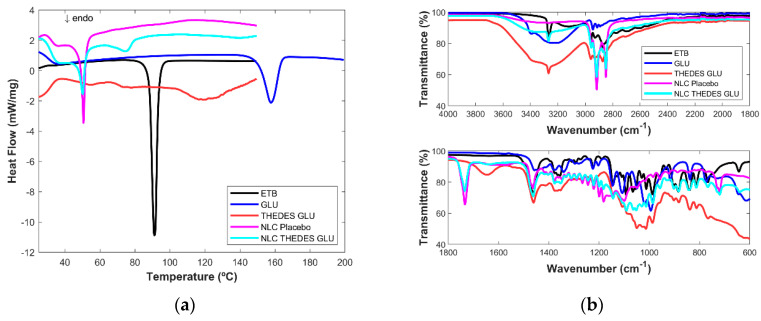
(**a**) DSC thermograms and (**b**) MIR spectra of ethambutol (ETB), glucose (GLU), ethambutol THEDES with glucose (THEDES GLU), NLCs placebo and the NLCs encapsulated with THEDES (NLC THEDES GLU).

**Figure 5:**
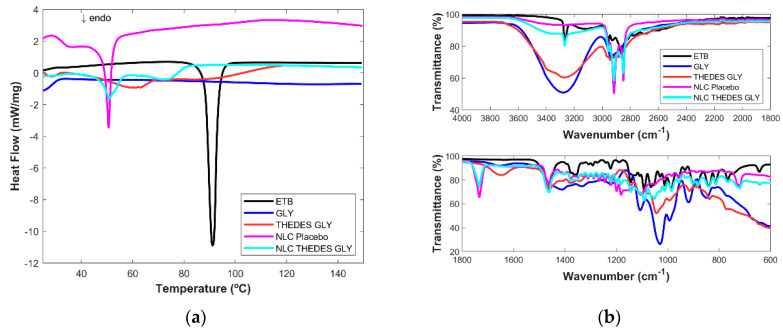
(**a**) DSC thermograms and (**b**) MIR spectra of ethambutol (ETB), glycerol (GLY), ethambutol THEDES with glycerol (THEDES GLY), NLCs placebo and the NLCs encapsulated with THEDES (NLC THEDES GLY).

For THEDES GLU (Figure 4a), the same depression can be seen between 60 °C and 80 °C; additionally, a very broad endothermic peak starting around 105 °C can be seen, indicating possible water evaporation. Regarding THEDES GLY (Figure 5a), a small endothermic peak can be seen between 50 °C and 70 °C. As already mentioned, this THEDES is not very stable, crystallizing easily; therefore, this peak can be the fusion of the THEDESs.

Unloaded NLCs made of cetyl palmitate have a main endothermic peak at an onset temperature of 49.3 °C and a peak temperature of 50.6 °C, and a smaller endothermic peak with an onset temperature of 30.4. °C and a peak temperature of 36.7 °C, which are related to the lipid polymorphic form and are in accordance with literature [25]. For all the NLC THEDES formulations, there is no endothermic melting peak of ETB which points to ETB dissolution into the lipid matrix, or, which is more likely in this case, ETB can be in the THEDES molecular structure, which does not also have an endothermic melting peak. Moreover, for all NLC THEDES, the DSC patterns show broadened, depressed endothermic peaks compared with the unloaded NLC, which indicates an increased number of lattice defects and disorder. Such behavior occurs when there are compounds dissolved in the lipid matrix, such as surfactant molecules, oils, or drugs [26].

In the case of NLC THEDES SCE, three endothermic peaks appear: the first one similar to the NLCs placebo with an onset of 25.3 °C and a peak temperature of 34.5 °C, ta second that can be deconvoluted in two, slightly enhanced compared to the unloaded NLC (T onset: 49.4 °C and peak temperatures of 52.4 °C and 53.9 °C), and a small one with a much higher temperature (65 °C). These can be attributed to distinct cetyl palmitate polymorphic forms depending on whether it is interacting or not with the THEDES SCE, indicating that there are influenced phases and non-influenced ones. Nevertheless, this increase in the temperature of the main endothermic peaks suggests the formation of more stable polymorphic forms that increase the formulations’ stability.

A very similar behavior is observed for NLC THEDES GLU: the first peak with an onset of 29.0 °C and a temperature of 36.4 °C; a second peak with an onset of 47.9 °C and a temperature of 50.2 °C and a third peak with an onset of 70.3 °C and a temperature of 74.5 °C. Only for NLC THEDES GLY, the presence of the main endothermic peaks like for the unloaded NLC (onset of 48.0 °C and a transition temperature of 50.9 °C) and an additional one, similar to those of the other NLC THEDES (onset of 64.8 °C and transition temperature of 72.9 °C), can be found.

Regarding the MIR results, for all the THEDESs (Figure 3b) (Figure 4b and Figure 5b), a large band between 3652 cm^−1^ and 2994 cm^−1^ and a smaller one at 1646 cm^−1^ can be seen due to the water present in the THEDES, and small peak at 3200 cm^−1^ due to ETB. This peak is not visible in the THEDES GLY (Figure 5b) since the band, due to the vibration of the OH group, is much larger in this case due to the intense spectral signature of glycerol in this region. From 1200 cm^−1^ and below, the spectrum is composed of broad and undefined bands characteristic of an amorphous compound.

In the case of the NLCs, the existence of the strong signal of ETB at 3264 cm^−1^ in all the NLC THEDESs leaves no doubt of its presence in all NLC formulations. NLCs encapsulated with THEDESs are very similar to each other. The main striking difference compared to unloaded NLC is the presence of the amount of water present in THEDES NLC (Figure 4b) which is visible due to a small, pronounced curve in the spectral region between 3600 cm^−1^ and 3100 cm^−1^. This observation points to the presence of THDESs in the NLC formulations and not ETB alone. Typical vibrations of the molecules present in the sugars are more difficult to assess due to the presence of many molecular vibrations. Nevertheless, the existence of water in the lyophilized NLC THEDES formulations leaves no doubt about the presence of THEDESs in NLC formulations.

## 3. Materials and Methods

### 3.1. Materials

Ethambutol hydrochloride (99% purity), glucose (>99% purity), sucralose (>98% purity), polysorbate 80, Dulbecco’s phosphate buffered saline, and dichloromethane (analytical grade) were purchased from Sigma Sigma-Aldrich (St. Louis, MO, USA). Cetyl palmitate was purchased from Gattefossé (Saint-Priest, France), miglyol 812 was acquired from Caelo (Hilden, Germany), sodium hydroxide was acquired from Merck (Darmstadt, Germany), and glycerol from LaborSpirit (Loures, Portugal). The water used was Milli-Q^®^ grade (resistivity of 18.2 MΩ·cm) purified by an Ultra-pure water system Heal Force (Shanghai, China). All chemicals were used without purification.

### 3.2. Ethambutol Preparation

Ethambutol hydrochloride (ETH) was used to obtain ethambutol (ETB) based on the method described by Bhutani [5]. Around 4 g of ETH was added to 40 mL of a 5 M aqueous solution of NaOH. The mixture was stirred for 15–20 min, and ETB was extracted with 40 mL of dichloromethane. The ethambutol crystals were obtained after slow evaporation in the fume hood at room temperature. Ethambutol was characterized by mid-infrared spectroscopy (MIR) and differential scanning calorimetry (DSC).

### 3.3. THEDES Preparation

After some preliminary tests, three THEDESs of ethambutol were obtained with sucralose (SCE), glucose (GLU), and glycerol (GLY); additionally, water was added to them in stoichiometric amounts. The following THEDESs were prepared (in parenthesis are the molar ratios): ETB:SCE:H_2_O (2:1:8), ETB:GLU:H_2_O (2:1:8), ETB:GLY:H_2_O (2:1:10). The THEDESs were prepared by weighing the three compounds at the given molar ratio into a glass vial that was sealed and placed in an incubator at 60 °C and 200 rpm for 2 h. After being removed from the incubator, the samples were allowed cooling at room temperature.

### 3.4. Mid Infrared Spectroscopy

For sample characterization, a mid-infrared spectrometer with Fourier Transform (Frontier PerkinElmer, Beaconsfield, UK) equipped with an attenuated total reflectance (ATR) accessory was used. The spectrum was obtained in the spectral region between 4000 and 600 cm^−1^, with 16 scan co-additions and 8 cm^−1^ of spectral resolution. To allow ideal contact between the sample and the crystal, the accessory ATR was equipped with a pressure arm with the indication of strength. The samples were applied directly to the ATR crystal and the same force was applied to each measurement, around 150. The instrument was controlled via Spectrum software (v. 10.03.09, PerkElmer, Beaconsfield, UK). The background was performed with the ATR empty.

### 3.5. Differential Scanning Calorimetry

DSC measurements were performed using a thermal analyzer (DSC 200 F3 Maia^®^, Netzsch, GmbH, Selb, Germany) with an automatic sample changer (ASC, Netzsch, GmbH, Selb, Germany). Approximately 7–10 mg was weighed in an aluminum pan and then sealed. The reference pan was left empty. Heating curves for the samples were recorded with a heating rate of 10 °C·min^−1^. Thermograms were analyzed using software provided by the DSC equipment (Proteus, version 6.1081, Netzsch, GmbH, Selb, Germany). The results presented are the average of at least two measurements.

### 3.6. Lipid Nanoparticles

Lipid nanoparticles were prepared by an optimized hot homogenization technique followed by a modified free organic solvent emulsification/sonification method [27]. NLC were prepared using miglyol-812 (50 mg) as the liquid lipid and cetyl palmitate (150 mg) as the solid lipid. Polysorbate 80 (50 mg) was added as the surfactant. After weighting the above-mentioned NLC components into a glass tube with the THEDESs corresponding to 200 mg of ETB, this mixture was placed in a water bath (70 °C), followed by an addition of 4 mL of water, also at 70 °C, when the mixture was fully melted. The suspension was placed under a probe sonicator for 5 min at 80% of intensity. The resulting nanoemulsion was stored in a sealed glass vial and left to cool at room temperature.

A portion of the encapsulated nanoparticles was frozen at −80 °C using a vertical ultra-arc Thermo-Heraeus (Waltham, MA, USA). After, the samples were lyophilized for 48 h under vacuum (0.4 mbar) using a Freeze Dryer LyoQuest (Telstar, Barcelona, Spain). The lyophilized samples were analyzed by DSC and MIR analysis.

#### 3.6.1. Size and Zeta Potential Assessment

Dynamic light scattering (DLS) was used to assess the nanoparticle hydrodynamic radius in the suspensions. To determine the hydrodynamic diameter and size distribution (polydispersiy index), a Particle Size Analyser (Brookhaven Instruments Corporation; Software: Particle Sizing v.5 Brookhaven Instruments; Holtsville, NY, USA) was used. The system was operating at a fixed light incidence angle of 90°, at 25 °C, with a dust cut-off set to 30. For each sample, a total of 6 runs for 2 min each was performed.

To determine the stability and morphology of the nanoparticles in suspension, the zeta potential was assessed using a Zeta Potential Analyser (ZetaPALS, Brookhaven Instruments Corporation; Software: PALS Zeta Potential Analyser v.5 Brookhaven Instruments; Holtsville, NY, USA). The system was operating at 25 °C, with a fixed light incidence angle of 90°. The mathematical model used for the analysis was Smoluchowski, and for each sample, a total of 6 runs, each one with 10 cycles, was performed.

#### 3.6.2. Encapsulation Efficiency (EE)

Encapsulation efficiency (EE) was determined by UV–Vis spectroscopy using a V-660 spectrophotometer (Jasco, B 034461152, Tokyo, Japan). Two quartz cuvettes of 1.4 mL (SMLQ-010-002) were used. The calibration curve was constructed with ETB concentrations between 0.01 and 0.09 mg/mL in ultrapure water at 203 nm. The diluted samples were transferred into Amicon^®^ Ultra-4 Centrifugal Filter Devices, ultracel^®^—50k (50,000 NMWL) (MERK Milipore, Ltd.; Cork, Ireland). Centrifugation was performed using an Allegra^®^ X-15R centrifuge (Beckman Coulter; Pasadena, CA, USA) until complete separation between NPs retained in the filter and the aqueous phase corresponding to the supernatant. The unentrapped ETB was present in the supernatant. The EE was calculated as follows (Equation (1)):

(1) $$EE\left(\%\right)=\frac{Total\;ETB\;amount-Unentrapped\;ETB}{Total\;ETB\;amount}\times 100.$$

Loading capacity (LC) was calculated using ETB encapsulation efficiency as follows (Equation (2)):

(2) $$LC\left(\%\right)=\frac{EE\times Total\;ETB\;amount}{Total\;lipid\;and\;surfactant\;amount}.$$

## 4. Conclusions

The development of THEDESs to improve the physicochemical properties of an API is in expansion; however, the inclusion of water in the THEDESs may lead to stability issues due to water evaporation and API crystallization. In the case of ETB, the API is already hygroscopic with stability issues. The new THEDESs can improve some of the problems of ETB, and its incorporation in NLCs can improve the stability of the THEDESs. The developed NLC THEDES formulations represent potentially effective delivery systems able to incorporate and retain the bioactive molecules while improving their stability, as shown by the DSC analysis and increased zeta potential values. DSC indicates the existence of more stable polymorphic forms of the lipid in the presence of THEDESs, and zeta potential values are more negative, which is evidence of surface modification.

The use of encapsulated THEDESs can bring new options to the treatment of tuberculosis since encapsulation can protect ethambutol from degradation and interaction with other anti-tuberculosis drugs. Moreover, the modification of the solid form of ethambutol from a crystalline salt to a deep eutectic liquid can also have a positive influence on the solubility and permeability of ethambutol, increasing the efficiency of the drug.

This work is a proof of concept on the use of NLCs to encapsulate a multicomponent liquid form instead of only incorporating the API alone. Encapsulation was achieved for the three THEDESs tested with formulations with a loading capacity superior to 8.5% and an encapsulation efficiency of between 43% and 47% Further studies will be developed to optimize the formulation for specific delivery pathways and to study this formulation in vitro.

## Figures and Tables

**Table 1:** Physicochemical characterization of the prepared NLCs.

Formulation	Hydrodynamic Radius (nm)	PDI	Zeta Potential (mV)	Encapsulation Efficiency (%)	Loading Capacity (%)
NLC (unloaded)	329 ± 6	0.08 ± 0.03	−33 ± 2	-	-
NLC THEDES SCE	413 ± 17	0.25 ± 0.07	−56 ± 2	43 ± 2	8.6 ± 2.0
NLC THEDES GLU	503 ± 27	0.24 ± 0.015	−51 ± 2	44 ± 2	9.3 ± 2.0
NLC THEDES GLY	492 ± 19	0.25 ± 0.016	−47 ± 2	47 ± 1	9.0 ± 1.0

## Data Availability

Data can be available upon request.
